# Neuronal splicing of the unmethylated histone H3K4 reader, *PHF21A*, prevents excessive synaptogenesis

**DOI:** 10.1016/j.jbc.2024.107881

**Published:** 2024-10-11

**Authors:** Masayoshi Nagai, Robert S. Porter, Maxwell Miyasato, Aijia Wang, Cecilia M. Gavilan, Elizabeth D. Hughes, Michael C. Wu, Thomas L. Saunders, Shigeki Iwase

**Affiliations:** 1 Department of Human Genetics, University of Michigan, Ann Arbor, Michigan, USA; 2 Genetics & Genomics Graduate Program, University of Michigan, Ann Arbor, Michigan, USA; 3 Neuroscience Graduate Program, University of Michigan, Ann Arbor, Michigan, USA; 4 University of Michigan College of Literature, Science, and the Arts, Ann Arbor, Michigan, USA; 5 Transgenic Animal Model Core, University of Michigan, Ann Arbor, Michigan, USA; 6 Neurodigitech, LLC, San Diego, California, USA; 7 Division of Genetic Medicine, Department of Internal Medicine, University of Michigan Medical School, Ann Arbor, Michigan, USA; 8 Department of Pediatrics, University of Michigan Medical School, Ann Arbor, Michigan, USA; 9 Michigan Neuroscience Institute, University of Michigan, Ann Arbor, Michigan, USA

**Keywords:** microexon splicing, histone methylation, histone demethylase, LSD1, PHF21A

## Abstract

PHF21A is a histone-binding protein that recognizes unmethylated histone H3K4, the reaction product of LSD1 histone demethylase. PHF21A and LSD1 form a complex, and both undergo neuron-specific microexon splicing. The PHF21A neuronal microexon interferes with nucleosome binding, whereas the LSD1 neuronal microexon weakens H3K4 demethylation activity and can alter the substrate specificity to H3K9 or H4K20. However, the temporal expression patterns of PHF21A and LSD1 splicing isoforms during brain development and their biological roles remain unknown. In this work, we report that neuronal PHF21A isoform expression precedes neuronal LSD1 expression during human neuron differentiation and mouse brain development. The asynchronous splicing events resulted in stepwise deactivation of the LSD1–PHF21A complex in reversing H3K4 methylation. An unbiased proteomics survey revealed that the enzymatically inactive LSD1–PHF21A complex interacts with neuron-specific binding partners, including MYT1-family transcription factors and post-transcriptional mRNA processing proteins such as VIRMA. The interaction with the neuron-specific components, however, did not require the PHF21A microexon, indicating that the neuronal proteomic milieu, rather than the microexon-encoded PHF21A segment, is responsible for neuron-specific complex formation. Finally, by using two *Phf21a* mutant mouse models, we found that *Phf21a* neuronal splicing prevents excess synapse formation that otherwise would occur when canonical PHF21A is expressed in neurons. These results suggest that the role of the PHF21A microexon is to dampen LSD1-mediated H3K4 demethylation, thereby containing aberrant synaptogenesis.

Brain development, the process in which the organ governing cognition forms, is arguably one of the most complex and intricate processes. Precisely regulated gene expression, including transcriptional regulation, is key for normal brain development. Human genetics studies of neurodevelopmental disorders have highlighted the critical roles of chromatin regulation in brain development. Chromatin regulators, which place, read, and erase histone and DNA modifications, represent a major gene group responsible for neurodevelopmental disorders, such as intellectual disability, schizophrenia, and autism spectrum disorders (1, 2, 3). However, chromatin regulators are broadly expressed across the body, making it challenging to understand the brain’s particular vulnerability to chromatin dysregulation.

Recent work has begun to unveil the unique features of chromatin regulations in neurons. For example, cytosine hydroxymethylation is most abundant in neurons and can play a role in gene regulation (4, 5). Non-CpG DNA methylation, primarily in the CA context, is found abundantly in neurons undergoing synaptogenesis and modulates synaptic gene expression (6, 7). In addition, our group has reported evolutionally conserved neuron-specific microexon splicing events in 14 chromatin regulators (8). In most cases, except for the LSD1 and PHF21A, as discussed below, the functional consequences of neuronal splicing events remain unknown. Thus, these observations have opened an area of research to investigate the roles and mechanisms of neuron-specific chromatin regulation and its potential link to human neurodevelopmental disorders.

Of the 14 chromatin factors that undergo neuron-specific splicing, two proteins—histone H3K4 demethylase LSD1 (aka KDM1A) and H3K4me0 reader protein PHF21A (aka BHC80)—are particularly intriguing for two reasons. First, their non-neuronal forms assemble into a stoichiometric complex suppressing neuron-specific genes in non-neuronal cells (9, 10). The complex consists of LSD1, PHF21A, class I histone deacetylases HDAC1 and HDAC2, HMG-box DNA binding protein BRAF35, and CoREST, which promote the nucleosome binding of LSD1 (9, 10, 11, 12, 13). REST/NRSF is the transcription factor that recruits the PHF21A–LSD1 complex to neuron-specific genes in non-neuronal cells for transcriptional silencing (9). Second, loss-of-function mutations in both genes, *KDM1A* and *PHF21A*, cause rare neurodevelopmental disorders that involve cognitive deficits (14, 15, 16). Since these mutations impact both canonical and neuronal isoforms of LSD1 and PHF21A, it remains to be determined which isoform is responsible for observed cognitive deficits.

The neuronal microexon splicing events in LSD1 and PHF21A cause small—just a few encoded amino acids—yet significant changes in their protein functions. The LSD1 neuronal splicing reduces H3K4 demethylation activity (17) and reportedly alters substrate specificity towards H3K9 (18) or H4K20 (19). Furthermore, the LSD1-Thr369, one of the four amino acids encoded by the neuronal microexon, is phosphorylated in the brain and, in turn, negatively modulates binding to CoREST, HDAC1/2 (20), and the nucleosome (8). The LSD1 neuronal splicing is modulated by neuronal activity (21), thereby contributing to excitation-inhibition balance, stress-response behavior (22), and learning and memory (19). Meanwhile, PHF21A neuronal splicing ablates the DNA-binding function of PHF21A mediated by an AT-hook motif, which is present in the canonical (PHF21A-c) but not in neuronal PHF21A isoform (PHF21A-n) (8). In sum, the emerging understanding is that the neuronal splicing events in LSD1 and PHF21A interfere with H3K4 demethylating function and contribute to the circuit homeostasis and cognitive functions.

Critical questions regarding LSD1 and PHF21A neuronal splicing still need to be addressed. First, developmental expression patterns of LSD1 and PHF21A isoforms have yet to be examined in detail. Second, we do not know whether neuronal LSD1 and PHF21A isoforms participate in a similar complex as non-neuronal cells or if the neuronal complex is unique. Third, the biological roles of PHF21A neuronal splicing remain unknown. To address these questions, in this study, we characterized the expression kinetics of LSD1 and PHF21A neuronal isoforms during neuronal differentiation, its consequences on the demethylase activity and complex formation, and examined the roles of PHF21A neuronal splicing in synaptogenesis.

## Results

### PHF21A completes switching to neuronal form prior to LSD1

**Figure 1:**
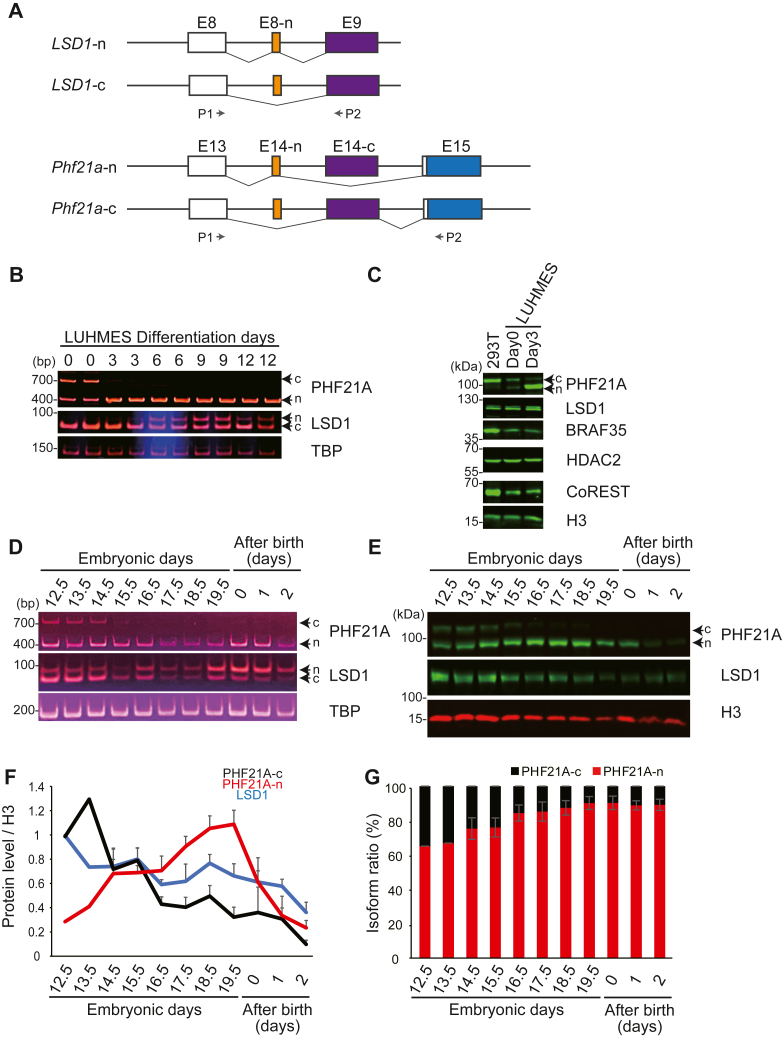
**The expression of PHF21A-n increases faster than that of LSD1-n.***A*, schematic representation of LSD1 and PHF21A neuronal splicing events. *B*, mRNA levels of LSD1 and PHF21A isoforms in LUHMES cells. Cells were differentiated into neurons as indicated and harvested from day 3 to day 12, analyzed by RT-PCR (n = 2). *C*, expression of PHF21A and associated proteins in LUHMES cells and 293T cells examined by Western blot analysis using antibodies as indicated. *D*, mRNA levels of LSD1 and PHF21A isoforms in the developing mouse brain. Whole-cell lysates prepared from mouse whole brains (E12.5 and E13.5) and cortices (from E14.5 to P2) at indicated periods were analyzed by RT-PCR. *E*, expression of PHF21A and LSD1 proteins in the developing mouse brain. Whole-cell lysates were subjected to Western blot analysis. *F*, quantification of Western signals for PHF21A-c, PHF21A-n, and LSD1 normalized by histone H3. PHF21A-c, and LSD1 levels were further normalized to E12.5. *G*, the ratio of PHF21A protein isoforms in the developing mouse brain based on the Western signals. *F* & *G*, day 12.5 to 13.5: n = 1, pooled embryonic brain is used. Day 14.5-P2: Mean ± S.E.M., n = 3, technical replicates.

The difference between PHF21A-n and PHF21A-c arises from the alternative usage of mutually exclusive exons, E14-n or E14-c (Fig. 1*A*). To examine the expression kinetics of PHF21A-n and LSD1-n during neuronal differentiation, we first turned to *in vitro* differentiation of Lund human mesencephalic (LUHMES) cells into neurons. LUHMES cells are known to produce a homogeneous neuronal population quickly once their differentiation is induced by dibutyryl-cAMP, thereby making them well-suited for biochemical investigations (23). We confirmed the homogenous morphological changes 3 days after the differentiation induction (Fig. S1*A*) and induction of neuronal markers, *RBFOX3* and *TUBB3*, as expected (Fig. S1*B*). The reverse transcriptase coupled polymerase chain reaction (RT-PCR) indicated that *PHF21A* mRNA completely switched from *PHF21A-c* to *PHF21A-n* after 3 days of neuronal differentiation (Fig. 1*B*). In contrast, the appearance of *LSD1-n* form began only after 6 days of differentiation. *LSD1-c* continues to be expressed during neuronal differentiation, which is consistent with the previous report (17).

To measure the *LSD1*-*n/c* ratio more quantitatively, we performed Illumina-based complete amplicon sequencing of the *LSD1* RT-PCR products harboring the alternative sequences internally. We adjusted the isoform ratio by accounting for the PCR efficiency difference; *LSD1-c* efficiency was 2.8% greater than *LSD1-n* PCR efficiency, which translates to a 1.62-fold underestimation of *LSD1-n* abundance, if uncorrected, in amplicon sequencing carried out with a 30-cycle PCR (Fig. S1, *C* and *D*, and see Experimental procedures). The LSD1-c:LSD1-n ratio obtained by the complete amplicon sequence was then adjusted with the correction values. *LSD1-n* emerged on day 3, kept increasing, and reached a plateau at approximately 37.7% on day 9 of LUHMES cell differentiations (Fig. S1*E*). A Western Blot analysis (WTN) using an anti-PHF21A antibody showed that the PHF21A-c protein was mostly replaced with PHF21A-n on day 3 (Fig. 1*C*), which agreed with the RT-PCR result. Note that WTN cannot distinguish LSD1-c and LSD1-n, which only differ in four amino acids; the doublet WTN signal originates from another alternative sequence located at the region close to the LSD1 N terminus (24). The large difference in *PHF21-n* and *PHF21A-c* PCR efficiency (23%) precluded us from a reasonably accurate quantification of the isoform ratio. These data indicated that the expression of PHF21A-n precedes that of LSD1-n in differentiating LUHMES cells.

Next, we examined the expression kinetics of the PHF21A and LSD1 isoforms in developing mouse brains. RT-PCR indicated that *PHF21A* mRNA largely switched from *PHF21A-c* to *PHF21A-n* after E15.5 mouse cortex (Fig. 1*D*). In contrast, the LSD1-c was present throughout the developmental periods examined. In this RT-PCR analysis, the LSD1-n/c ratio appeared to show stepwise increases on E16.5 and P0. The quantification of the results with the Illumina amplicon sequencing indicated that *LSD1-n* mRNA consists of 42% at E18.5 and increased further to 81% at P0 brain (Fig. S1*E*). In contrast, the percentage of PHF21A-n protein reached a plateau (91%) already in the E16.5 mouse cortex (Fig. 1, *E*–*G*) like *PHF21A* mRNA isoforms. Total protein levels of LSD1 and PHF21A decreased after birth, implicating a greater contribution of these factors to embryonic brain development. These results indicate that, similar to the observation in LUHMES cells, the increase of neuronal form is faster for PHF21A than LSD1 *in vivo*. Since PHF21A adopts the neuronal form earlier than LSD1, the two proteins potentially form a complex containing PHF21A-n and LSD1-c prior to the mature neuronal complex containing PHF21A-n and LSD1-n, especially in the late gestation period.

### The complex with PHF21A-n and LSD1-c demethylates H3K4

**Figure 2:**
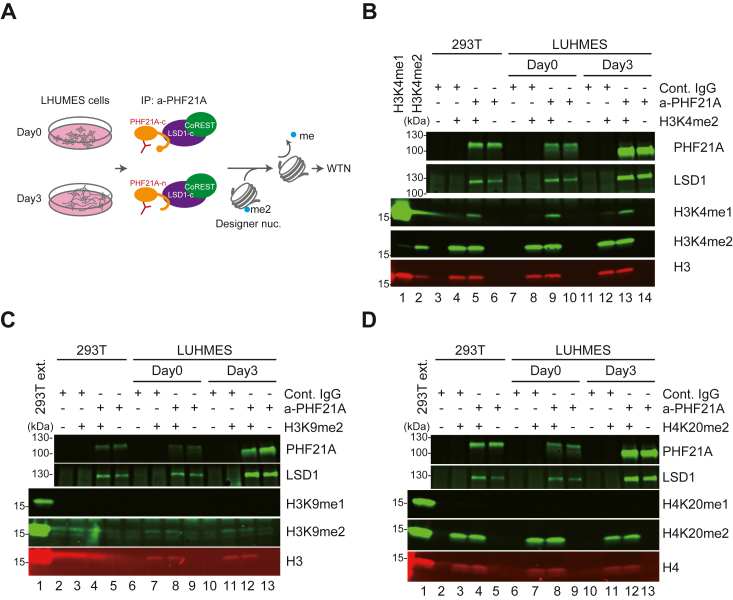
**A weaker H3K4 demethylation activity of the PHF21A-n:LSD1-c complex.***A*, schematic of the demethylation assay. The PHF21A-containing complex was immunoprecipitated from LHUMES cell nuclear extracts and incubated with designer nucleosomes with specific lysine di-methylations. *B*-*D*, Western blot using the antibodies for indicated histone methylations to detect demethylase activity. The reactions were carried out using H3K4me2- (*B*), H3K9me2- (*C*), and H4K20me2- (*D*) nucleosomes. The appearance of mono-methylated lysine indicates the demethylation activity. The abundance of LSD1, PHF21A, and total H3 (c-term) were examined with specific antibodies. H3K4me1/me2 designer nucleosomes and 293T nuclear extract serve as specificity and positive control for the histone antibodies, respectively.

Prior studies have described LSD1-n as a weaker H3K4 demethylase (17), H3K9- (18), and H4K20- (19) demethylase. Having established the differential expression kinetics of PHF21A and LSD1 isoforms, we sought to determine the substrate specificity of the PHF21A-n:LSD1-c complex. We immunoprecipitated (IP) the complexes with a PHF21A antibody from undifferentiated (day 0) and differentiating (day 3) LUHMES cells. PHF21A-IP samples from both day 0 and day 3 contained LSD1, indicating that PHF21A-n can interact with LSD1 (Fig. 2). On day 0, 62.8% of PHF21A and 100% of LSD1 adopt the canonical form (Fig. 1*B* and S1*E*). On day 3, 91.6% of PHF21A adopts the neuronal form (Fig. 1*B*), while 94% of LSD1 is still the canonical form (Fig. S1*E*). Thus, the major complexes at day 0 and day 3 were PHF21A-c: LSD1-c and PHF21A-n: LSD1-c, respectively.

We then incubated the IP samples with recombinant designer mono-nucleosomes carrying either H3K4me2, H3K9me2, or H4K20me2. Demethylation reactions can be detected by either the decrease of dimethylation or the appearance of monomethylation after the reaction *via* Western blot analysis, as previously described (Fig. 2*A*) (8). As a control, we examined the enzymatic activity of PHF21A complex from 293T cells, which only express PHF21A-c and LSD1-c (Fig. 2, 293T lanes). We reliably detected the generation of H3K4me1 by the PHF21A complexes isolated from both day 0 and day 3 LUHMES cells as well as 293T cells (Fig. 2*B*, quantified in Fig. S2, *A* and *B*). The H3K4me1 signals were absent or nearly absent when the H3K4me2-nucleosome was omitted from the reaction, ruling out the coprecipitated cellular H3K4me1-nucleosome as the WTN signal source (Fig. 2*B*, lanes 6, 10, &14). The control IgG IP samples did not show any activity (Fig. 2*B*, lanes 4, 8, and 12). In this LUHMES cell system, the overall abundance of PHF21A was greater during differentiation than the undifferentiated state, leading to a greater LSD1 level in IP samples, although the total LSD1 level was unchanged during differentiation (Fig. 1*C*). With the greater LSD1 level, the day 3-complex yielded a similar level of H3K4me1 compared to the day 0-complex, suggesting weaker enzymatic activity of the PHF21A-n:LSD1-c complex than the canonical PHF21A-c:LSD1-c complex.

With H3K9me2 or H4K20me2 nucleosomes, we did not find either a decrease in the di-methylation signal or the appearance of mono-methylation, indicating that the complexes were inactive on these substrates (Fig. 2, *C* and *D*). Cellular fractionation assays using LUHMES cells showed that the percentages of chromatin-bound PHF21A and LSD1 were unchanged during the differentiation, in which PHF21A-c converts into PHF21A-n (Fig. S2, *C* and *D*). These results demonstrate that H3K4 is the substrate of the PHF21A-n:LSD1-c complex.

### No detectable demethylation activity of the mature neuronal complex with PHF21A-n and LSD1-n

In our previous work with reconstituted LSD1–CoREST–PHF21A tripartite complex, we found that the mature neuronal complex with LSD1-n and PHF21A-n has weaker H3K4 demethylase activity and lacks detectable activity to H3K9me2 and H4K20me2 (8). However, the reconstitution experiment with purified proteins left a possibility that additional interaction partners could bestow new substrate specificity to the neuronal complex. Indeed, a previous report showed that the interaction with SVIL protein in neurons led LSD1-n to demethylate H3K9 instead of H3K4 (18). We, therefore, sought to determine the substrate specificity of the neuronal complex with PHF21A-n and LSD1-n isolated from neurons.

Our initial attempt was to differentiate LUHMES cells further than day 3 to the point where a significant fraction of LSD1 adopts neuronal form. However, the LSD1-n level did not reach >40% and reached a plateau on day 9, and subsequent culture did not result in a greater LSD1-n ratio, as discussed earlier (Fig. S1*E*). We also noted that prolonged culture of LUHMES cells led to fewer cell numbers and made it impractical to collect sufficient IP materials for demethylation assays. For these reasons, we turned to the mouse P0 brains, which show high expression levels of the two proteins adopting predominantly neuronal forms; 94% of PHF21A and 81% of LSD1 are neuronal forms (Figs. 1*G* and S1*F*).

**Figure 3:**
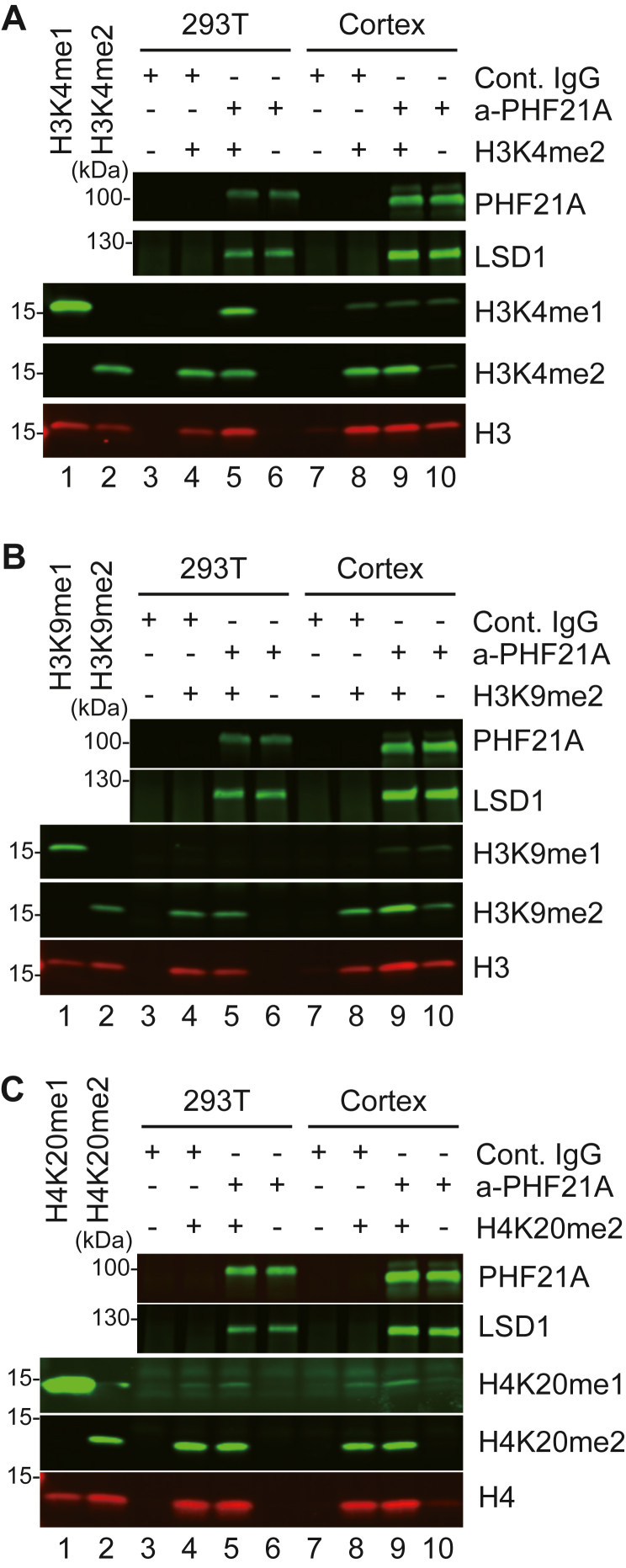
**No detectable demethylation activity of the mature neuronal complex with PHF21A-n and LSD1-n.** The demethylation assay of immunoprecipitated PHF21A complexes from the P0 mouse cortices using the designer nucleosomes carrying H3K4me2 (*A*), H3K9me2 (*B*), and H4K20me2 (*C*). Neither a reduction of di-methylation nor the appearance of mono-methylation was observed with any demethylation reactions.

We carried out PHF21A IP from the P0 mouse cortices and demethylation assay using the recombinant designer nucleosomes (Fig. 3, *A*–*C*). Unlike the PHF21A-n:LSD1-c complex in Fig. 2, we did not detect the specific appearance of H3K4me1 when both the H3K4me2 nucleosomes and the complex were present in the reaction (Fig. 3*A*, compare lane 9 to lanes 8 & 10). Meanwhile, the PHF21A complex from 293T cells shows the specific H3K4me1 appearance (Fig. 3*A*, lane 5) in the parallel reactions. Importantly, LSD1 levels in PHF21A-IP samples were greater in the brain samples than 293T, ruling out the possibility that an insufficient amount of LSD1–PHF21A complex resulted in the lack of enzymatic activity. When we used the H3K9me2 and H4K20me2 nucleosomes, the P0 brain PHF21A complex did not consistently generate mono-methylations either (Fig. 3, *B* and *C*). These results indicate that the mature neuronal complex with LSD1-n and PHF21A-n isolated from the brain lacks detectable enzymatic activity in these assays.

### Comparative proteomics analysis of PHF21A-containing complex in MEFs and neurons

**Figure 4:**
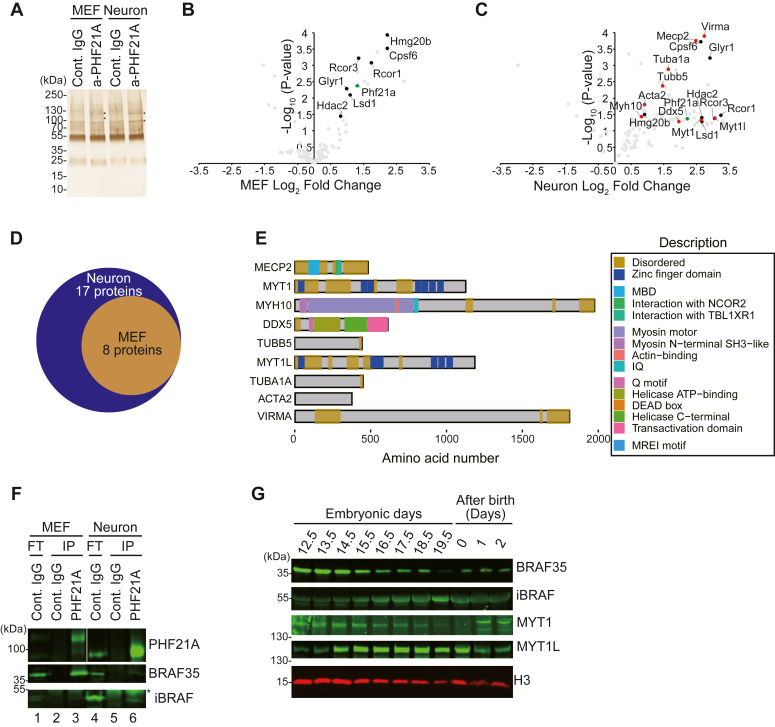
**Proteomics analysis of PHF21A-containing complex in MEFs and neurons.***A*, silver staining of proteins co-precipitated by an anti-PHF21A antibody. Nuclear extracts from MEFs and cortical neurons (DIV7) were used. Asterisks indicate proteins specifically found in PHF21A-immunoprecipitates. *B*-*C*, Co-IP-MS analysis of PHF21A-associated proteins in MEF (*B*) and cortical neurons (*C*). Volcano plots of identified proteins (n = 3). *Green* dots: the bait protein, PHF21A. *Black* and *red* dots: the interactors (Cutoff: log2FC > 1.5, *p*-value < 0.1, and peptide ≥ 6). *Red* dots: neuron-specific interactors with the same cut-off. *Gray* dots: proteins that did not pass the cutoff. The x-axis shows the log_2_ FC (a-PHF21A antibody/control IgG), and the y-axis denotes -log_10_*p*-values. *D*, Venn diagram of PHF21A interactor in MEFs (*orange*) *versus* neurons (*blue*). *E*, domain organization of neuron-specific PHF21A interactors. *F*, Co-IP-Western assays to test the interaction between PHF21A, BRAF35, or iBRAF. Asterisks indicate nonspecific IgG bands. *G*, expression of neuron-specific PHF21A interactors in the developing mouse brains. Whole-cell lysates prepared from mouse whole brains (E12.5 and E13.5) and cortices (from E14.5 to after birth 2) at indicated periods were subjected to Western blot analysis using antibodies as indicated. The H3 blot was re-used from Fig. 1*E*.

**Figure 5:**
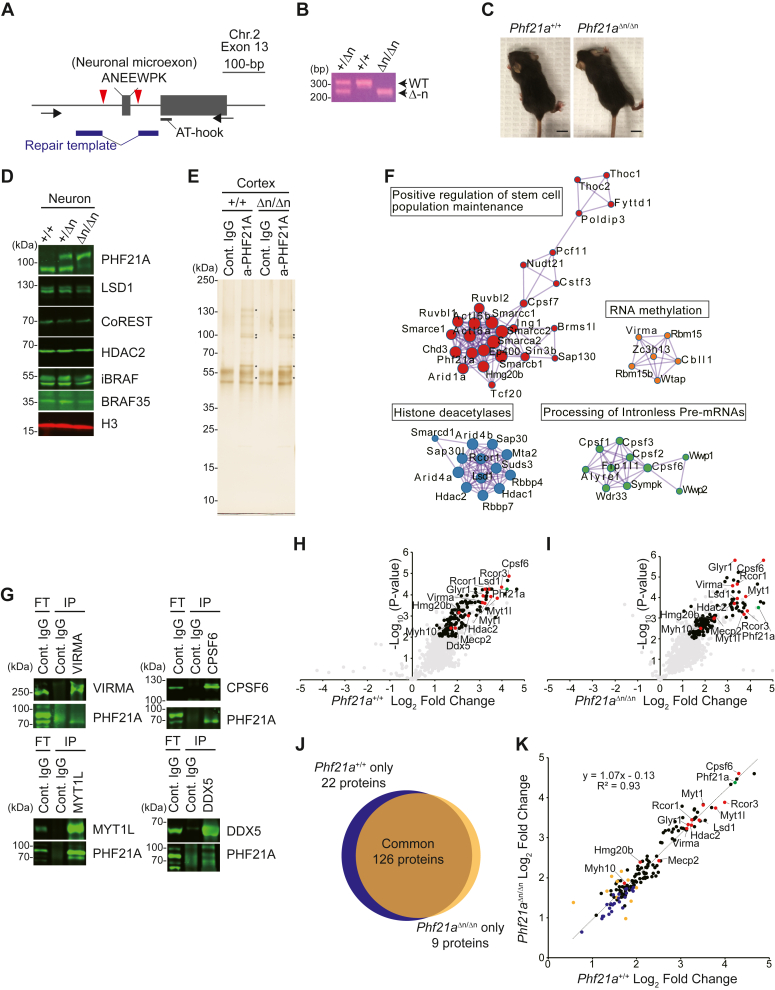
**Binding partners of PHF21-n and ectopic PHF21A-c in neurons are highly similar.***A*, generation of *Phf21a*-neuronal exon KO allele (Δn). *Black* arrows: PCR primers. *Red* triangles: CRISPR cut sites. *B*, genotyping PCR analysis of *Phf21a*^+/+^, *Phf21a*^+/Δn^, and *Phf21a*^Δn/Δn^ mice using the primer sets shown in (*B*). *C*, representative picture of P15 *Phf21a*^+/+^ (*left*) and *Phf21a*^Δn/Δn^ (*right*) mice. Scale bar represents 1 cm. *D*, Western blot analysis of lysates prepared from cortical neurons (DIV7) isolated from *Phf21a*^+/+^, *Phf21a*^+/Δn^, and *Phf21a*^Δn/Δn^ mice using antibodies as indicated. PHF21A-c instead of PHF21A-n is expressed in *Phf21a-n*^Δn/Δn^ neuron. *E*, silver staining of proteins co-precipitated by anti-PHF21A antibody using the nuclear extracts from P0 cortices of the indicated genotypes. Asterisks indicate proteins specifically found in PHF21A-immunoprecipitates. *F*, functional enrichment analysis of PHF21A-interacting proteins with Metascape (41). The statistically significant PHF21A interactors in *Phf21a*^+/+^ cortices were used. Log_10_*p*-value < −10. The molecular networks that contain proteins validated by reciprocal IP experiments are presented. *G*, reciprocal Co-IP-Western assays to validate the interaction between PHF21A and newly identified interaction partners. *H* and *I*, volcano plots of Co-IP-MS analysis of PHF21A-interacting proteins (n = 3–4) using *Phf21a*^+/+^ (*H*) and *Phf21a*^Δn/Δn^ (*I*) cortices. *Green* dots: the bait protein, PHF21A. *Black* and *red* dots: statistically significant interactors (Cutoff: FC > 2, *Padj*-value < 0.01, and peptide ≥ 6). *Red* dots: neuron-specific proteins identified in Fig. 4*C* that pass the cutoff. *Gray* dots: proteins that do not pass the threshold. The x-axis shows the log_2_ FC (a-PHF21A antibody/control IgG), and the y-axis denotes -log_10_*p*-values. *J*, Venn diagram of PHF21A interactor in *Phf21a*^+/+^ cortex (*blue*) *versus Phf21a*^Δn/Δn^ cortex (*orange*). *K*, scatter plot comparing the log2FC (a-PHF21A antibody/control IgG) of all PHF21A-associated proteins between WT and *Phf21a*^Δn/Δn^ cortices. The R^2^ value and the fit of the linear regression are indicated. *Green* dots: the bait protein, PHF21A. *Black* and *red* dots: statistically significant interactors in *Phf21a*^+/+^ a-PHF21A and *Phf21a*^Δn/Δn^ (Cutoff: *Padj*-value < 0.01, and peptide ≥ 6). *Red* dots: neuron-specific proteins identified in Fig. 4*C* that pass the cutoff. *Blue* dots: statistically significant interactors in *Phf21a*^+/+^ a-PHF21A. *Orange* dots: statistically significant interactors in *Phf21a*^Δn/Δn^. The x-axis shows the log_2_ FC of *Phf21a*^+/+^ (a-PHF21A antibody/control IgG). The y-axis shows the log_2_ FC of *Phf21a*^Δn/Δn^ (a-PHF21A antibody/control IgG).

We then reasoned that investigating PHF21A-n–interacting proteins in neurons might provide insights into the undetectable demethylation activity of the mature complex. To this end, we performed a comparative PHF21A immunoprecipitation coupled with mass spectrometry (IP-MS) study using mouse embryonic fibroblast (MEF) and cortical neuron cultures (DIV7) (Fig. 4*A*). LSD1-n is 91% of total LSD1 in the cortical neurons, while absent in MEF (Fig. S1*G*). PHF21A is predominantly the canonical form in MEF and neuronal form in cortical neurons (Fig. 4*F*). Though proteins specifically precipitated by the PHF21A antibody were barely visible in silver staining (Fig. 4*A*), we were able to identify consistently coprecipitated proteins across replicates (n = 3) (Table S1). Of the total 108 proteins identified, eight proteins in MEF and 17 proteins in cortical neurons passed our threshold: *p* < 0.1, FC (anti-PHF21A/control IgG) > 1.5, and peptide number ≥ 6 (Fig. 4, *B* and *C*). Notably, all eight proteins identified in MEF were also identified in neurons (Fig. 4*D*). Most of these common interactants, such as LSD1, RCOR1 (aka CoREST), HDAC2, and HMG20B (aka BRAF35), were known components of previously isolated LSD1 complexes (9, 10, 25, 26). In neurons, nine additional proteins were identified. Of these, zinc-finger transcription factors MYT1 and MYT1L are exclusively expressed in embryonic neurons (27) (Fig. 4*E*). Primary roles of some neuron-specific PHF21A-interaction partners are outside chromatin regulation; DDX5 and VIRMA have been implicated in splicing and mRNA methylation (28, 29, 30, 31, 32, 33), while TUBB5, TUBA1A, ACTA2, and MYH10 comprise of the cytoskeleton (34, 35, 36, 37), which required further validation of interaction (See Fig. 5).

BRAF35/HMG20B is a component of the canonical PHF21A–LSD1 complex purified from non-neuronal HeLa cells (9, 10). Previous work showed that a BRAF35 paralogue called iBRAF (aka. HMG20A) is expressed in mature neurons instead of BRAF35 (38, 39). iBRAF inhibits the action of BRAF35, thereby promoting the expression of neuron-specific genes (38, 39). These observations led us to postulate that BRAF35 interacts with PHF21A-c while iBRAF interacts with PHF21A-n. Since our MS analysis was inconclusive due to the short polypeptides of BRAF35 and iBRAF, we tested whether BRAF35 and iBRAF interact with PHF21A isoforms by co-immunoprecipitation Western analysis. We found that BRAF35 was expressed in both MEF and neurons, whereas the iBRAF was primarily expressed in neurons (Fig. 4*F*, lanes 1&4). In MEF, BRAF35 was co-immunoprecipitated with PHF21A-c as expected, while in neurons, iBRAF was co-immunoprecipitated with PHF21A-n. Notably, though BRAF35 expression levels are comparable between MEF and neurons, the co-immunoprecipitated BRAF35 level was much lower in neurons than in MEF (Fig. 4*F*, lanes 3&6). These results suggest that BRAF35 can interact with PHF21A-n; however, iBRAF outcompetes BRAF35 for PHF21A-n binding in neurons.

Having identified neuron-specific PHF21A-interacting proteins, we sought to understand the temporal dynamics of PHF21A-containing complexes during brain development. To this end, we examined the expression kinetics of these novel PHF21A partners in developing mouse brains by Western blot analyses. Consistent with the previous reports (38, 39), BRAF35 expression decreased during brain development, which is accompanied by the concomitant increase of iBRAF expression (Fig. 4*G*). Such reciprocal expression pattern was also found in MYT1 and MYT1L; the MYT1 level was higher in early development (E12.5 to E15.5), whereas MYT1L started increasing from E14.5. After birth, however, the expression of BRAF35 and MYT1 returned, while their pairs, iBRAF and MYT1L, plunged their expression again, representing an intricate regulation of these complex components. Together, these results suggest that PHF21A interacts with both canonical and neuron-specific proteins, and the interaction partners can change during neuronal differentiation owing to their dynamic expression patterns.

Unlike the previous report with SH-SY5Y cells (18), we did not detect SVIL protein in the neuronal PHF21A complex, which might explain why we did not detect the H3K9 demethylation activity of the complex (Fig. 3*C*). Another work from our group demonstrates that neuronal splicing of PHF21A and LSD1 both contribute to the reduced H3K4 demethylation activity of the complex by interfering with their contacts with the nucleosomes (8). However, the reconstituted neuronal complex with LSD1-n, CoREST, and PHF21A-n still exhibited H3K4 demethylase activity, albeit weaker than its canonical counterpart (8). A plausible explanation of the difference is that the reconstituted complex in the other study was much more abundant than the complex from the brain used in the present study. Nonetheless, these observations all agree with the negative impact of the two neuronal splicing events on the enzymatic activity of the LSD1–PHF21A complex.

### The binding partners of PHF21A-n and ectopic PHF21A-c in neurons are highly similar

Having identified new neuron-specific PHF21A-binding partners, we wondered if being the neuronal form enables PHF21A to interact with these new partners in neurons. To address this question, we have generated a mouse model in which the *Phf21a* neuronal microexon is deleted by CRISPR-Cas9 (The *Phf21a*-Δn allele, Fig. 5, *A* and *B*). In contrast to *Phf21a*-KO mice, which lack both canonical and neuronal forms and die neonatally (40), homozygous *Phf21a*-Δn mice (*Phf21a*^Δn/Δn^) were viable (Fig. 5*C*). In the cortical neurons isolated in *Phf21a*^Δn/Δn^ mice, PHF21A-c is expressed at a comparable level with PHF21A-n in WT (*Phf21a*^+/+^) neurons (Fig. 5*D*). Thus, *Phf21a*-Δn represents the swapping mutant, in which PHF21A-n is replaced with PHF21A-c in neurons, allowing us to test if being neuronal form is necessary to interact with neuronal binding partners.

First, we examined whether the ectopic PHF21A-c expression in neurons alters the stability of binding partners for which specific antibodies are available. We did not observe any overt changes in the levels of LSD1, CoREST, HDAC2, BRAF35, and iBRAF, indicating that ectopic PHF21A-c does not impact the stability of known binding partners (Fig. 5*D*). In addition, overexpression of PHF21A-n, PHF21A-c, LSD1-n, or LSD1-c did not change the protein levels of endogenous PHF21A, LSD1, BRAF35, HDAC2, CoREST, and stably-expressed iBRAF in 293T cells (Fig. S3*A*). Thus, neuronal PHF21A and LSD1 splicing events do not influence the stability of the binding partners examined.

Next, we performed proteomics quantification of PHF21A-associated proteins in *Phf21a*^+/+^ and *Phf21a*^Δn/Δn^ mice cortex (P0) with IP-MS (n = 3–4 per genotype). As discussed earlier, at this stage of brain development, 94% of PHF21A and 81% of LSD1 are neuronal forms in *Phf21a*^+/+^ mice cortex (Figs. 1*G* and S1*F*). Thus, *Phf21a*^+/+^ data mostly represents PHF21A-n–associated factors in neurons, and *Phf21a*^Δn/Δn^ data represents PHF21A-c–associated factors in neurons. In this P0 brain proteomics study, unlike the earlier study with DIV7 cortical neuron cultures (Fig. 4*A*), we saw clear protein bands unique to PHF21A-IP samples (Fig. 5*E*) and identified many more proteins (149 in *Phf21a*^+/+^ samples, padj < 0.01, FC > 2, and peptide number ≥ 6) (Table S2). Proteins identified in DIV7 mostly overlapped with the proteins found in the P0 brain (Fig. S3*B*). The greater numbers of identified proteins are likely due to higher PHF21A levels in the P0 brain than in DIV7 neurons. With Metascape analysis (41), we found these PHF21A-interacting proteins belong to several protein-interaction networks: positive regulation of stem cell population maintenance, HDACs deacetylate histones, processing of intronless pre-mRNAs, and RNA methylation (Fig. 5*F* and Table S3). Reciprocal IP assays validated some of these new PHF21A-interaction partners, including VIRMA, CPSF6, MYT1L, and DDX5 (Fig. 5*G*).

The proteomic profile of the *Phf21a*^Δn/Δn^ mice cortex sample was highly similar to that of *Phf21a*^+/+^ (Fig. 5, *H* and *I*). In the *Phf21a*^Δn/Δn^ mice cortex samples, we identified 126 proteins, which largely overlapped with those found in WT samples (Fig. 5*J*). Twenty two proteins were uniquely found in WT, and nine proteins were uniquely found in *Phf21a*^Δn/Δn^. To determine whether these unique binding factors between WT and *Phf21a*^Δn/Δn^ samples reflect genuine changes or borderline statistical significances, we compared the PHF21A-Ab/control IgG fold enrichment values (log2FC, the average of 3–4 replicates) of all PHF21A-interacting proteins found in either *Phf21a*^+/+^ or *Phf21a*^Δn/Δn^ samples (Fig. 5*K*). The log2FC enrichment values show a high concordance (R^2^ = 0.93) between genotypes, and the linear regression slope was 1.07. The log2FC values of these *Phf21a*^+/+^- and *Phf21a*^Δn/Δn^-specific binding proteins still show high concordance between genotypes, and these proteins mostly had low FC values. Furthermore, differential enrichment analysis between PHF21A-Ab–bound proteins between *Phf21a*^+/+^ and *Phf21a*^Δn/Δn^ yielded no statistically significant proteins (Fig. S3*C*). Thus, we concluded that interacting proteins did not substantially change in neurons when PHF21A-n was replaced with PHF21A-c.

These results demonstrate that PHF21A participates in unexpected molecular networks, such as post-transcriptional regulation, in neurons, and the PHF21A neuronal splicing is dispensable for the interaction between PHF21A and its neuronal partners.

### The roles of neuronal splicing in synaptogenesis

The above molecular studies led to the question of why neurons generate the hypomorphic neuronal PHF21A isoform. To address this question, we employed two *Phf21a* mutant mouse models. The first model harbors the constitutive *Phf21a*-null allele (40). Homozygous *Phf21a*-null mice (*Phf21a*^−/−^) lack both neuronal and canonical isoforms (40). The second model is the above *Phf21a*-Δn mice, in which PHF21A-c replaces PHF21A-n in the neurons. We examined synaptogenesis in the two mouse models because synaptic deficits are common in neurodevelopmental disorders associated with chromatin dysregulations (42).

**Figure 6:**
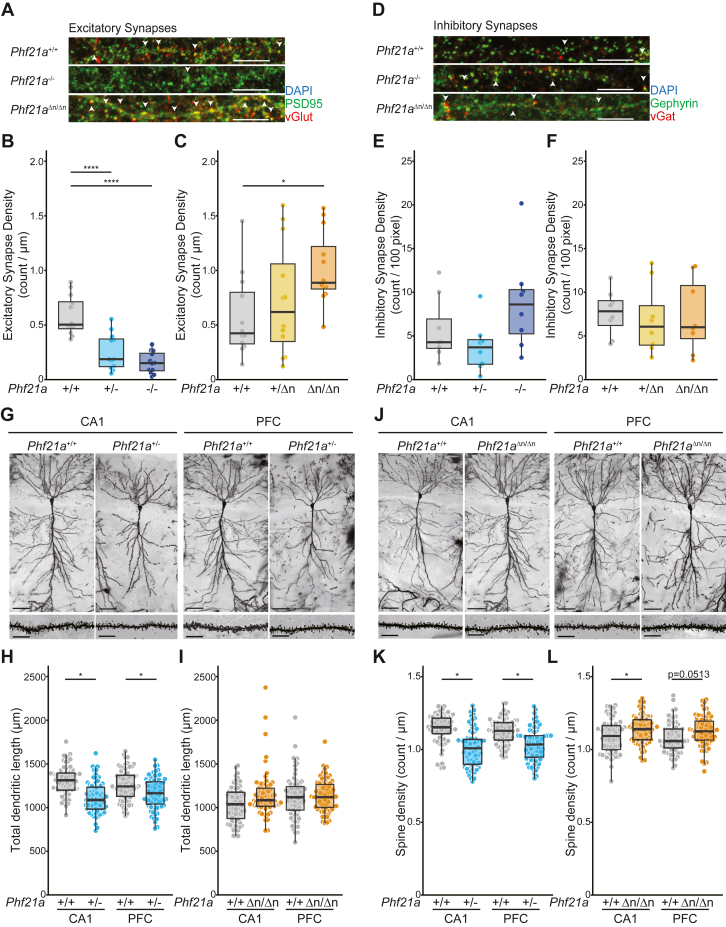
**Characterization of synapse and dendrite growth in *Phf21a*-mutant mice.***A*-*F*, synapse density in cultured cortical neurons. Cortical neurons from E16.5 embryos (2–6 per genotype) were cultured for 14 days *in vitro* (DIV14). *A* and *D*, the representative images of excitatory synapses (*A*), visualized by the immunofluorescent colocalization of postsynaptic marker PSD95 (*green*) and presynaptic marker vGlut (*red*), formed on cortical pyramidal neurons. Inhibitory synapses (*D*) were visualized by the colocalization of postsynaptic Gephyrin (*green*) and presynaptic vGat (*red*). Nuclei were stained with DAPI (*blue*). Scale bars represent 10 μm. *B*-*F*, quantification of excitatory (*B* and *C*) and inhibitory (*E* and *F*) synapse density in *Phf21a*-null (*B* and *E*) and *Phf21a*-Δn (*C* and *F*) mutants (mean ± S.E.M., n = 12, ∗*p* < 0.05, ∗∗∗∗*p* < 0.001, one-way ANOVA with Tukey *post hoc* analysis). *G*-*L*, dendritic morphology analysis of CA1 and PFC pyramidal cells of *Phf21a* mutants. *G* and *J*, the representative Golgi staining images of CA1 and PFC pyramidal cells of *Phf21a*^+/+^, *Phf21a*^+/−^, and *Phf21a*^Δn/Δn^ mice. The *upper* panel shows the dendritic morphology of pyramidal cells, and the *lower* panel shows the dendritic spines of a dendritic segment. Scale bars represent *upper*: 100 μm, *lower*: 5 μm. *H* and *I*, quantification of dendritic length in *Phf21a*-null (*H*) and *Phf21a*-Δn (*I*) mutants (n = 72). *K* and *L*, quantification of dendritic spine density in *Phf21a*-null (*K*) and *Phf21a*-Δn (*L*) mutants (n = 72). The boxes show the *lower* and *upper* quartile values, respectively. Both apical and basal dendrites from 12 neurons per animal were quantified: three of 2-month-old males per each genotype were used. The median is indicated with a bar within the box, and the whiskers denote the 1.5 × data range of the box. (∗*p* < 0.05, ∗∗∗∗*p* < 0.001, Unpaired Student’s *t* test).

First, we quantified the synaptic density in the primary cultures of cortical neurons (Fig. 6, *A*–*C*). Excitatory synapses were visualized by the colocalized immunofluorescent signals of pre- and post-synaptic markers on the dendritic shafts of pyramidal neurons. We observed a significant decrease of excitatory synapses in *Phf21a*-null neurons, in which heterozygous mutant neurons exhibit an intermediate phenotype between WT and homozygous mutant neurons (Fig. 6, *A* and *B*). In contrast, excitatory synapse density was higher in *Phf21a*^Δn/Δn^ neurons (Fig. 6, *A* and *C*). Inhibitory synapse densities did not change either in *Phf21a*^−/−^ or *Phf21a*^Δn/Δn^ neurons (Fig. 6, *D*–*F*).

Next, we quantified the dendritic growth and dendritic spine density *in vivo* by Golgi staining; dendritic spines are the post-synaptic structure of excitatory synapses (Fig. 6, *G*–*L*). We chose two brain regions, the hippocampal CA1 and prefrontal cortex (PFC), essential for cognitive functions. For the *Phf21a*-null model, we only analyzed heterozygous mutants (*Phf21a*^+/−^) because homozygous mutants die immediately after birth prior to major synaptogenesis. In the *Phf21a*^+/−^ neurons, the dendritic lengths of pyramidal neurons were significantly shorter than WT in both CA1 and PFC (Fig. 6, *G* and *H*). *Phf21a*^Δn/Δn^ neurons exhibited slight upward shifts of dendritic length, but the shifts were not statistically significant (Fig. 6*I*). Consistent with the results of cultured neurons, the dendritic spine density showed significant decreases in *Phf21a*^+/−^ neurons, while an increase in *Phf21a*^Δn/Δn^ neurons, in CA1. The PFC dendritic spines showed similar results, except that the difference between WT and *Phf21a*^Δn/Δn^ neurons did not reach statistical significance (Fig. 6, *G* and *L*).

These results indicate that PHF21A plays an important role in excitatory synapse development. When PHF21A-c replaced PHF21A-n in neurons, more excitatory synapses formed, suggesting that PHF21A-n is synthesized to prevent excess synapse formation mediated by PHF21A-c.

## Discussion

In this work, we have described the asynchronous neuronal splicing of LSD1 and PHF21A in differentiating human neurons and developing mouse brains. The lagged LSD1 neuronal splicing implicated a stepwise transition of the complex abundance from LSD1-c:PHF21A-c, LSD-c:PHF21A-n, and then LSD1-n:PHF21A-n during brain development. The resulting complexes are deactivated for H3K4 demethylating activity along with this transition. Our proteomics studies revealed that PHF21A-n interacts with the known proteins and new neuron-specific partners in neurons. Unexpectedly, the neuronal splicing of PHF21A was dispensable for interacting with these neuron-specific binding partners. Neuronal morphological analyses of the two *Phf21a* mutant mouse lines indicated that the microexon prevents excess synaptic formation.

The substrate specificity of LSD1-n has been shown as H3K4 (8, 17), H3K9 (18), and H4K20 (19). In the present study, similar to our prior study (8), we did not detect H3K9 or H4K20 demethylating activity in either the PHF21A-n:LSD1-c or PHF21A-n:LSD1-n complexes. Instead, we observed the stepwise deactivation of the complex, which ultimately became incapable of reversing H3K4me in our assays. The lack of activity towards H3K9 can be explained by the absence of SVIL protein in our PHF21A-IP samples. We do not have any reasonable explanation for the lack of activity toward H4K20, given that this activity was observed in a purified system involving recombinant CoREST and LSD1 isoforms (19). It could be argued that H4K20 demethylation is not the function of LSD1-n that forms a complex with PHF21A; our complex was purified by an anti-PHF21A antibody rather than an anti-LSD1 antibody. However, others and our prior work did not detect H4K20 demethylation activity using the bipartite complex of recombinant LSD1-n and CoREST either (8, 43). To resolve this issue fully, it is imperative to identify the responsible variables between these observations and seek additional evidence, such as X-ray crystallography.

Regardless of the LSD1-n neofunctionalization issue, the common conclusion agreed by all the studies is that LSD1 neuronal splicing impairs its H3K4 demethylation activity. In the present study, our observations indicate that the developing brain relentlessly decommissions the LSD1–PHF21A complex and might make the complex play distinct functions by interacting with unique proteins in neurons. For example, we identified neuron-specific zinc-finger transcription factors MYT1 and MYT1L as neuron-specific PHF21A-binding proteins (Fig. 4). MYT1 and MYT1L belong to Myt-transcription factor family and are expressed specifically in neurons with particularly high levels in the embryonic brain (44). Both MYT1 and MYT1L bind to a consensus DNA motif AAAGTTT (27) and transcriptionally suppress the Notch signaling pathway, which negatively regulates neurogenesis, in turn, promotes neurogenesis (45, 46). MYT1L also represses the transcriptional program of deeper layer neurons in the adult prefrontal cortex (47) and non-neuronal genes (46, 48). SIN3-HDAC is a major corepressor that enables MYT-mediated transcriptional suppression in vertebrates (47, 49). However, an MYT1 proteomics study with Neuro2A cells identified the LSD1–CoREST–PHF21A complex (50), which is consistent with our results and suggests plausible roles of the complex in the MYT-mediated transcriptional regulation of the neurodevelopmental program. Furthermore, MYT1L can also activate the transcription of neuronal maturation genes with unknown mechanisms (27, 51). It is tempting to speculate that the neuronal splicing of LSD1 and PHF21A is responsible for the functional switch of MYT1L from repressor to activator, with the neuronal LSD1–PHF21A complex acting as a dominant negative machinery. Lastly, this is the first report describing the interaction between the LSD1–PHF21A complex and post-transcriptional mRNA processing machinery such as CPSF6 and VIRMA. The association of these mRNA processing proteins is specific or much stronger in neurons (Figs. 4 and 5). While CPSF6 controls alternative polyadenylation (52), VIRMA promotes m6A RNA methylation (53). Thus, the neuronal LSD1–PHF21A complex might be involved in mRNA processing in neurons. Further investigations are needed to understand this process better.

Lastly, our work provides the initial clue to the question: why does PHF21A need to adopt neuronal form in neurons? Excitatory synapse numbers increased in a PHF21A functional dose-dependent manner, where *Phf21a*-null cells showed the least synaptic density, while neurons with *Phf21a*-c showed the greatest synaptic density both *in vitro* and *in vivo* (Fig. 6). The results suggest that PHF21A can promote gene expression program of synaptogenesis; however, PHF21A-c is too potent in this function. Functional dampening of PHF21A by microexon splicing allowed optimal synapse numbers, which might have provided selective advantages by optimizing cognitive functions during vertebrate evolution. Interestingly, the PHF21A-n does not achieve the optimal synaptic density by providing unique protein–protein interactions, given that the protein interactome of PHF21A-c in neurons was essentially identical to that of PHF21A-n (Fig. 5). Future studies should address how PHF21A-n working with newly identified interacting partners in confining the synapse numbers within a proper range.

## Experimental procedures

### Cell culture

#### LUHMES cells

LUHMES cells were purchased from ATCC (CRL-2927). The culturing and handling procedures of LUHMES cells were as described previously (54). In brief, LUHMES cells were grown at 37 °C in a 5% CO_2_ atmosphere in DMEM/F-12 (Gibco 11,330–032) containing N2 supplement (Gibco 17502048) and 40 ng/ml bFGF (Peprotech 100-18B). Before plating, plates were coated with PDL overnight at 37 °C and washed with water thrice. Confluent cultures were passaged by trypsin digestion. One day after plating, the proliferation medium was replaced with DMEM/F-12 containing N2 supplement, 1 μg/ml tetracycline (Sigma T7660), 1 mM dibutyryl cyclic AMP (Selleck Chemicals S7858) and 2 ng/ml Recombinant Human GDNF (Peprotech 450–10).

#### HEK293T

HEK293T cells were grown at 37 °C in a 5% CO_2_ atmosphere in DMEM (Gibco 11995065), supplemented with 10% FBS. Primary Neurons: The primary neuron culture was performed as previously described (8) with slight modifications. For molecular analyses, Cytarabine (10 μ M, aka AraC, Tocris) was added to the culture at DIV3 to eliminate the non-neuronal cell growth. MEFs: MEFs culture was performed as previously described (8). Cells were passaged less than twice for most experiments.

### Antibodies and PCR primers

Primary antibodies used are the following: rabbit anti-PHF21A (1:1000, in house, raised against 1–100 aa of human PHF21A expressed and purified from E.coli), rabbit anti-LSD1 (1:1000, Abcam ab17721), rabbit anti-BRAF35 (1:1000, ABclonal A4408), rabbit anti-HDAC2 (1:200, Santa Cruz sc-7899), rabbit anti-CoREST (1:1000, Abcam ab183711), mouse anti-H3 (1:500, Santa Cruz sc-517576), rabbit anti-H3K4me1 (1:5000, Abcam ab176877), rabbit anti-H3K4me2 (1:5000, Revmab bioscience 31–1037–00), rabbit anti-H3K9me1 (1:5000, Epicypher 13–0029), rabbit anti-H3K9me2 (1:5000, Abcam ab194680), rabbit anti-H4K20me1 (1:2500, Abcam ab9051), rabbit anti-H4K20me2 (1:5000, Abcam ab9052), mouse anti-H4 (1:200, Abcam ab31830), rabbit anti-iBRAF (1:1000, ABclonal A7286), rabbit anti-Myt1 (1:500, Invitrogen PA5-85510), rabbit anti-Myt1l (1:500, Proteintech 25234-1-AP) and rabbit anti-MeCP2 (1:500, Proteintech 10861-1-AP), Virma (1:1000, 25712-1-AP), CPSF6 (1:1000, ab99347), DDX5(1:2000, ab126730). The primers used for the amplification are as follows: hLSD1_F1: GCTGTGGTCAGCAAACAAG, hLSD1_R3: ATATTCCTTGCATAGGGCGGTC, hLSD1_F2: CCCACTTTATGAAGCCAACGGAC, hLSD1_R2: CAACCGGTTAAACTCTTGCTCTACC, hPHF21A_F1: GCAGTGACATACCTAAACAGC, hPHF21A_R1: CCAGGATGGTGTTCTTCATTTC, hTBP_1: GATCTTTGCAGTGACCCAGC, hTBP_2: CGCTGGAACTCGTCTCACTA, hTUBB3_for: GTGAAAACTGCGACTGCCTG. hTUBB3_rev: ACGACGCTGAAGGTGTTCAT, hRBFOX3_for: GCATGACCCTGTACACACCA, hRBFOX3_rev: TTCTCTGTAGGGTCGGAGGG, mLSD1_F3: CCCACTTTATGAAGCCAATGGAC, mLSD1_R3: CAACCGGTTAAATTCTTGTTCTACC, mLSD1_F1 AGGTCTTGGAGGGAATCCCATG, mLSD1_R1 ATTCCTTGCAGAGGGCAGTC, mPHF21A_F1: CAGTCACTTACCTTAACAGCAC, mPHF21A_R1: TGCTGCTCTTCATCTCCATAC, mTbp_qPCR_F: TTCAGAGGATGCTCTAGGGAAGA, mTbp_qPCR_R: CTGTGGAGTAAGTCCTGTGCC, Phf21a-n-F2: ACAGACGCCCAGCACCTTTAG, Phf21a-n-R2: GTAAGGGCTCCAAACCCCAG.

### RNA purification and complete amplicon sequence

Total RNA was isolated using the RNeasy Plus Mini Kit (QIAGEN 74134) according to the manufacturer’s protocol. The RNA concentration was determined using a BioSpectrometer (Eppendorf). Total RNA (1 μ g) was used to generate the ProtoScript II First Strand cDNA Synthesis Kit (Biorad E6560S) following the manufacturer’s instructions. PCR reactions were performed with KOD Hot Start DNA Polymerase (Sigma 71086–3) using a Mastercycler X50a (Eppendorf). Complete amplicon sequencing was performed by the CCIB DNA Core Facility at Massachusetts General Hospital (Cambridge, MA). Illumina-compatible adapters with unique barcodes were ligated onto each sample during library construction. Libraries were pooled in equimolar concentrations for multiplexed sequencing on the Illumina MiSeq platform with 2 × 150 run parameters. Upon completion of the NGS run, data were analyzed, demultiplexed, and subsequently entered into an automated *de novo* assembly pipeline, UltraCycler v1.0 (Brian Seed and Huajun Wang, unpublished).

### Quantitative RT-PCR

The expression of different target genes was validated by quantitative PCR (qPCR) using the 7500 Real-Time PCR Systems (Applied Biosystems). The reactions were performed with the Power SYBR Green PCR Master Mix (Applied Biosystems 4367659) as recommended by the manufacturer. Real-time PCR was performed with a hot start step of 50 °C for 2 min and 95 °C for 10 min, followed by 30 cycles of 95 °C for 15 s, 60 °C for 1 min, and analyzed with 7500 software (Applied Biosystems). The PCR efficiency was calculated by linear regression between Ct values and concentrations of human LSD1-c and LSD1-n cDNA in pENTR plasmids. The resulting PCR efficiencies (*E*) were 73.5% for LSD1-c and 70.8% for LSD1-n, where 100% defines a doubling of DNA copy number per one PCR cycle. The correction value was calculated as $\left[\left(E_{\text{LSD}1-c}+1\right)ˆ30/\left(E_{\text{LSD}1-n}+1\right)ˆ30\right]$. The LSD1-c: LSD1-n ratio obtained by the complete amplicon sequence described above was multiplied by the LSD1-n value with the correction value (1.62).

### Cell fractionation and WTN analysis

LUHMES and 293T cells were fractionated by Subcellular Protein Fractionation Kit (#78840, Thermo Scientific). Whole-cell lysates were prepared from mouse whole brains (E12.5 and E13.5) and cortices (E14.5 to P2). Cells were lysed in radioimmunoprecipitation assay (RIPA) buffer (50 mM Tris-HCl pH7.5, 150 mM 5M NaCl, 0.5% Sodium Deoxycholate monohydrate, 0.1% sodium dodecyl sulfate (SDS) and 1% TritonX-100) supplemented with Protease Inhibitor (Roche, 11873580001). Extracted proteins were boiled for 10 min with 2X Laemmeli buffer (100 mM Tris-HCl pH6.8, 4% SDS, 0.2% Bromophenol blue, 20% Glycerol and 5% β-mercaptoethanol) at 100 °C. Proteins were separated by SDS-polyacrylamide gel electrophoresis (PAGE) electroblotted onto a polyvinylidene difluoride (PVDF) membrane (Millipore, IPFL00010). PVDF membrane was masked with Intercept Blocking Buffer (LI-COR, 927–70001) for 2 h at 4 °C, incubated with primary antibodies in Intercept Blocking Buffer overnight at 4 °C, washed with PBST (137 mM NaCl, 2.7 mM KCl, 11.9 mM phosphates and 0.1% Tween20) and incubated with secondary antibodies in Intercept Blocking Buffer for 1 h at RT. After the final washes, fluorescence signals were detected using Odyssey DLx Imager (LI-COR).

### Co-immunoprecipitation analysis (Co-IP)

Nuclei were enriched from the Dounce homogenized 293T cells, MEFs, neurons, and cortex using EZ Nuclei Lysis buffer (10 mM Tris at pH 7.4, 10 mM NaCl, 5 mM MgCl2, 0.5% NP-40, x1 Protease inhibitor cocktail). Nucleoproteins were extracted from nuclei with the same volume of IP Extraction Buffer (20 mM HEPES at pH 7.9, 1.5 mM MgCl_2_, 0.6 M KCl, 0.2 mM EDTA, 0.5 mM DTT, 25% Glycerol, x1 Protease inhibitor cocktail) as EZ Nuclei Lysis buffer for 30 min at 4 °C. For Co-IP, samples were bound to 5ug of crosslinked antibody (Rabbit IgG, a-PHF21A) each sample for 3 h at 4 °C. Then, samples were subjected to SDS-PAGE and Western blot analyses. For Co-IP WTN, to reduce the background signals originating from IgG molecules used for IP, the PHF21A antibody was biotinylated by Pierce Antibody Biotinylation Kit (90407) following the manufacturer’s protocol and used as the primary WTN antibody. Likewise, for PHF21A IP, antibodies (Control rabbit IgG, a-PHF21A) were crosslinked as follows: first, the antibodies were reacted with Protein A/G beads (1:1 mixture) overnight at 4 °C, then crosslinked by 10 μ M DMP (Thermo 21667) in 0.2 M sodium borate pH 9.0 for 1 h at RT. The reaction was quenched with 0.2 M Tris-HCl pH 8.0 at room temperature for 1.5 h, and the antibody-conjugated beads were washed with IP buffer and used for IP reaction.

### Demethylation assays

The PHF21A-containing complexes were immunoprecipitated with 50 ug of crosslinked antibody for 3 h at 4 °C. One ug of recombinant designer demethylated nucleosomes (EpiCypher) was incubated with the PHF21A-containing complexes from 293T cells, LUHMES cells, or the mouse cortices for 3.5 h at 37 °C in histone demethylation buffer (50 mM Tris-HCl at pH 8.0, 50 mM KCl, 0.5% BSA, 5% glycerol, 0.5 mM DTT). The demethylation activity was measured by the appearance of monomethylation in WTN using specific methyl-histone antibodies listed above.

### Co-IP-MS

The immunoprecipitated protein was isolated from MEF cells and DIV7 neurons of E16.5 or P0 mouse cortices with the abovementioned in-house PHF21A antibody. Rabbit IgG from unimmunized rabbits (Jackson ImmunoResearch) was used as a control. pus (n = 3∼4). The PHF21A-complex was immunoprecipitated with 40 ug of a-PHF21A antibody as described above, eluted by 0.1% trifluoroacetic acid, and neutralized by 200 mM EPPS (4-(2-hydroxyethyl) piperazine-1-propane sulfonic acid) buffer. Then, samples were added to a 1:1 mixture of 2X Laemmeli buffer boiled for 10 min at 100 °C. Proteins were separated on a 4 to 20% SDS-PAGE gel (Biorad 4561096, 5671095) and visualized with a Piece Silver Stain kit (Thermo 24612) according to the manufacturer’s protocol. The samples were multiplex with isobaric tandem mass tags (TMT) and analyzed with Orbitrap Fusion Tribrid Mass Spectrometer at Thermo Fisher Scientific Center for Multiplexed Proteomics at Harvard Medical School (Cambridge, MA). Gene ontology (GO) annotation analysis was performed using Metascape (41).

### *Phf21a* exon 13 knockout (*Phf21a-Δn*) mouse

CRISPR/Cas9 technology was used to introduce double-strand breaks upstream and downstream of *Phf21a* exon 13. The mouse *Phf21a* gene (ENSMUSG00000058318) is located on the forward strand of Chromosome two from nucleotides 92,014,451 to 92,195,011 (GRCm39). Loss of exon 13 will cause an out-of-frame mRNA with multiple premature termination codons expected to trigger nonsense-mediated mRNA decay (55). The CRISPOR algorithm (56, 57) was used to identify highly specific single guide RNAs (sgRNA) in introns 12 and 13: intron 12 sgRNA (C189C): 5′-AAGGTTAATACACAGGCCAG (PAM = AGG)-3’ (CFD score of 85). Intron 13 sgRNA (C189Y): 5′- AAAATGATCTTACATACCTT (PAM = TGG)-3′, (CFD score of 69). Phosphorothioate-modified sgRNA was synthesized by Synthego (58, 59). Each sgRNA (30 ng/ul) was complexed with enhanced specificity (eSP) Cas9 protein (50 ng/ul from Millipore-Sigma (60) and individually validated for causing chromosome breaks in mouse zygotes. The ribonucleoproteins (RNPs) were microinjected into fertilized mouse eggs. Eggs were placed in culture until they developed into blastocysts. DNA was extracted from individual blastocysts for analysis. PCR with primers spanning the predicted cut sites was used to generate amplicons for Sanger sequencing (61). To test sgRNAs C189C and C189Y, a 668-bp amplicon was produced with forward primer: 5′- GTCTGAACTGTTAGCAAAGAGACACAGAAA-3′; and reverse primer 5′-AGAGAGTACATGTCCCCAAGTTACTTAC-3′. Sequencing electropherograms of amplicons from individual blastocysts were evaluated to determine if small insertions/deletions caused by non-homologous end joining (NHEJ) repair of chromosome breaks were present (62). Using high-specificity sgRNA and enhanced specificity Cas9 protein dramatically reduces the likelihood of off-target hits in mice (63). The CRISPR reagents were microinjected into fertilized mouse eggs produced by mating superovulated C57BL/6J female mice (Jackson Laboratory stock no. 000664) with C57BL/6J male mice as described (64). CRISPR/Cas9 microinjection of zygotes produced founder mice. Generation zero founder (G0) pups were identified by Sanger sequencing of the PCR amplicons spanning the expected deletion. G0 founders were mated with wild-type C57BL/6J mice to obtain germline transmission of the *Phf21a* mutant allele. *Phf21a-Δn* mice were backcrossed into C57BL/6J for at least five generations to remove off-target mutations.

### Mice

We bred the animals in groups of one or two under specific pathogen-free conditions (*ad libitum* access to food and water, 12:12 light: dark cycle, light on at 06:00 AM). Mice were tagged using ear punch and randomly assigned to each experiment. Timed-pregnant CD-1 mice were purchased from Charles River to obtain E16 embryos and P0 pups. All animal use followed NIH guidelines and approved by the University of Michigan Committee on Use and Care of Animals.

### Expression plasmids

PHF21A-c, PHF21A-n, LSD1-c, and LSD1-n cDNAs cloned into pENTR-D-TOPO, the Gateway Entry System (Invitrogen) entry vector, were previously described (8). Full-length coding sequences of BRAF35 and iBRAF were synthesized (Twist Bioscience) and cloned into pENTR-D-TOPO. To prepare expression plasmids, the cDNA fragments in pENTR-D-TOPO were transferred to the modified pHAGE plasmids (8) using LR recombination (Invitrogen 56,484).

### Establishment of a stable cell line expressing iBRAF and transfection

pHAGE plasmid containing iBRAF was co-transfected with psPAX2 and pMD2.G into 293T cells as previously described (65). After 2 days of passaging, one ug/ml of puromycin was added to the medium and selected for >3 days. HEK293 T cells were transfected with expression plasmids carrying PHF21A-c, PHF21A-n, LSD1-c, LSD1-n, BRAF35 or iBRAF cDNAs using Lipofectamine 3000 reagent (Invitrogen) for 24 h and harvested.

### Primary culture of mouse forebrain neurons

The primary culture was performed as previously described (Garay *et al*., 2020). Timed pregnant female mice were sacrificed at day E16.5, and embryonic brains were harvested. The cortices and hippocampi were microdissected, treated with 2.5% Trypsin (Invitrogen, 15,090), quenched by FBS, and then treated with 1% DNase (Sigma, DN-25) dissociate brain tissue into neurons. Cells were counted and then plated in Neurobasal Media (Gibco, 21103049) with 1x B27 (Gibco, 17504–044), 0.5 mM GlutaMax (Gibco, 21985), 25 uM beta-mercaptoethanol, and 1% Penicillin-Streptomycin. Before plating, plates were coated with Poly-D-lysine hydrobromide (PDL, Sigma P7886, mw 30000–70000) overnight at 37 °C and then washed with ddH2O three times. Neuron culture cells were fed every 3 to 4 days by replacing half of the above media with new media.

### Synapse quantification

Forebrain neurons were grown on PDL-coated coverslips for 14 days without Cytarabine. Then, cells were washed in artificial CSF (145 mM NaCl, 3 mM KCl, 1 mM CaCl2, 1 mM MgCl2-6H2O, 8 mM dextrose, 10 mM HEPES), fixed in paraformaldehyde, quenched by 0.01 M glycine, washed in PBS, blocked in 10% BSA in PBS, and then incubated with the following primary antibodies overnight at 4 °C. Excitatory synapse markers were PSD95 (NeuroMab K28/43, 1:500) and vGlut (Synaptic Systems 135303, 1:1000). Inhibitory synapse markers were used Gephyrin (Synaptic Systems 147111, 1:500) and vGat (Synaptic Systems 131004, 1:1000). Slides were washed three times in 3% BSA in PBS, incubated with secondary antibodies (1:1000) along with DAPI (1 ug/ml), washed three more times, and then mounted on slides using ProLong Gold Antifade Mountant (Invitrogen P36930). Slides were blinded to genotype and imaged using a Nikon A1 inverted confocal microscope. Images were taken in parts of the slide where the number of neuron cell bodies was equal. Laser levels, microscope settings, and subsequent thresholding were kept equal across all samples. ImageJ was used to separate channels and threshold the images (66). Then NeuronJ plugin was next used to trace dendrite segments (67). Finally, the SynapCountJ plugin was used to assess colocalization of pre- and postsynaptic puncta along the defined dendrites, and average synapse density per 100 pixels was reported (68).

### Golgi staining and dendritic morphology analyses

The Golgi staining, cryosectioning, imaging, and analyses were performed as previously described (69). Brains from 2-months mice, three male animals per genotype, were dissected and incubated in a modified Golgi-Cox solution for 2 weeks at room temperature. Pyramidal neurons in the frontal cortex and dorsal hippocampus CA1 per animal were quantified: N = 12 neurons from each animal were measured. For each neuron, one apical and one basal dendrite are quantified. Quantification was done using commercially available software, NeuroLucida (v10, Microbrightfield, VT), installed on a Dell PC workstation that controlled a Nikon Eclipse Ni microscope with Hamamatsu CCD camera (C11440, ORCA-Flash4.0) (Full Resolution: 2048 pixels × 2048 pixels), motorized X, Y, and Z-focus for high-resolution image acquisition (×100 oil immersion) and quantifications. The morphological analyses included dendritic lengths, spine counts, and overall spine density. All sample genotypes were blinded to the analysts throughout the course of the analysis. After completion, the digital profile of neuron morphology was extrapolated and transported to a multi-panel computer workstation, then quantitated using NeuroExplorer program (Microbrightfield, VT).

## Data availability

The row data supporting this study's findings are available in University of Michigan Deep Blue data (https://doi.org/10.7302/y4bw-4n85).

## Supporting information

This article contains supporting information (41).

## Conflict of interest

M. C. W. is the CEO of Neurodigitech, LLC. The other authors declare that they have no conflicts of interest with the contents of this article.
