# Supplementary material for: Neuronal splicing of the unmethylated histone H3K4 reader, PHF21A, prevents excessive synaptogenesis
Source: J Biol Chem. 2024 Oct 11;300(11):107881. doi: 10.1016/j.jbc.2024.107881 (PMC11605454; doi:10.1016/j.jbc.2024.107881)

## Title

Neuronal splicing of the unmethylated histone H3K4 reader, *PHF21A,* prevents excessive synaptogenesis

## Authors

Masayoshi Nagai^1,a^, Robert S. Porter ^2,b^, Maxwell Miyasato^3^, Aijia Wang^4^, Cecilia M. Gavilan^2^, Elizabeth D. Hughes^5^, Michael C. Wu^6^, Thomas L. Saunders^5, 7^, and Shigeki Iwase^1,8,9†^

1. Department of Human Genetics, University of Michigan, Ann Arbor, MI, 48109, USA.
2. Genetics & Genomics Graduate Program, University of Michigan, Ann Arbor, MI, 48109, USA.
3. Neuroscience Graduate Program, University of Michigan, Ann Arbor, MI, 48109, USA.
4. University of Michigan College of Literature, Science, and the Arts, Ann Arbor, MI, 48109, USA.
5. Transgenic Animal Model Core, University of Michigan, Ann Arbor, MI 48109, USA.
6. Neurodigitech, LLC, San Diego, CA 92126, USA
7. Division of Genetic Medicine, Department of Internal Medicine, University of Michigan Medical School, Ann Arbor, MI, 48109, USA.
8. Department of Pediatrics, University of Michigan Medical School, Ann Arbor, MI 48109, USA.
9. Michigan Neuroscience Institute, University of Michigan, Ann Arbor, MI 48109, USA.

† To whom correspondence should be addressed: [siwase@umich.edu](mailto:siwase@umich.edu)

a. Present address: Department of Medical Biochemistry, Graduate School of Medicine, Osaka Metropolitan University, Osaka 545-8585, Japan.

b. Present address: Department of Neurology, Mass General Brigham, Boston, MA, 02114, USA.

**Supporting Information:**

- **Supporting Figure 1: LUHMES cell differentiation and PCR efficiency of LSD1 isoforms.**
- **Supporting Figure 2: Quantification of H3K4me after demethylation assays and chromatin binding of PHF21A-LSD1 complex.**
- **Supporting Figure 3: Impact of overexpressing PHF21A interactors on the stability of other complex components and additional analyses of the proteomics data.**
- **Supporting Table 1: Co-IP-MS analysis of PHF21A-associated proteins in MEF and cortical neurons.**
- **Supporting Table 2: Co-IP-MS analysis of PHF21A-associated proteins in *Phf21a*^+/+^ and *Phf21a*^Δn/Δn^ cortices.**
- **Supporting Table 3: Functional enrichment analysis of PHF21A-interacting proteins with Metascape.**

## Supporting Figure Legend

**Supporting Figure 1: LUHMES cell differentiation and PCR efficiency of LSD1 isoforms.**

**(A)** Morphology of LUHMES cells at day 0 and day 3 of differentiation. Scale bar, 100 μm. **(B)** RT-pPCR analyses for the neuronal markers *RBFOX3* (NEUN) and *TUBB3* validate the neuronal differentiation of LUHMES cells (mean ± S.E.M., n=3). **(C)** PCR efficiency of LSD1 mRNA isoforms empirically determined by the LSD1-c and LSD1-n cDNA-carrying plasmids. The values were averaged from three technical replicates at four concentrations. **(D)** Correction values of LSD1 isoform expression after 30 cycles using the efficiency difference determined in **(C)**. LSD1-c is estimated to have a 1.62-fold higher expression than RT-qPCR measurement after the correction. **(E)** The ratio of LSD1-c and LSD1-n mRNA isoforms. Cells were differentiated into neurons as indicated and harvested on day 3 to day 12. Data were adjusted for PCR efficiency, which was determined in (C). **(F & G)** The ratio of LSD1-c and LSD1-n mRNA isoforms in the developing mouse brain (mean ± S.E.M., n=3, panel F) and cultured MEF or cortical neurons (panel G). Data were adjusted for PCR efficiency, which was determined in (C).

**Supporting Figure 2: Quantification of H3K4me after demethylation assays and chromatin binding of PHF21A-LSD1 complex.**

**(A & B)** The WTN signals of H3K4me1 **(A)** and H3K4me2 **(B)** were quantified by the LICOR imager after demethylation reaction with immunoprecipitated PHF21A complex from LUHMES cells (mean±data range, n=2). **(C)** Cellular fractionation assays were used to evaluate the chromatin binding of the PHF21A-LSD1 complex at day 0 and day 3 of LUHMES cell differentiation. WTN was performed with the indicated antibodies. **(D)** Quantification of Western signals for **(C)**. The WTN signals were quantified by the LICOR imager (mean ± S.E.M., n=3, n.s.= not significant, unpaired Student’s t-test).

**Supporting Figure 3: Impact of overexpressing PHF21A interactors on the stability of other complex components and additional analyses of the proteomics data.**

**(A)** The protein levels of PHF21A-interactors, LSD1, iBRAF, BRAF35, HDAC2, and CoREST were examined after overexpressing the indicated proteins in 293T cells by Western blot analysis. 293T cells do not express iBRAF, so we generated a stable cell line expressing iBRAF and examined the impact of overexpression of the above molecules on the iBRAF protein level. **(B)** Overlap of the PHF21A-interacting proteins identified from cortical neurons (Fig. 4) and neonatal brains (Fig. 5). **(C)** Volcano plot comparing the proteins immunoprecipitated by PHF21A antibody from *Phf21a*^+/+^ vs. *Phf21a*^Δn/Δn^ brains. No protein showed significant differences between the genotypes.

**Supporting Table 1: Co-IP-MS analysis of PHF21A-associated proteins in MEF and cortical neurons.**

The complete list of identified proteins in Co-IP-MS comparing MEF and cortical neurons. Raw MS values (summed S/N values scaled to 100 per protein) and statistics, including -log(P), and log2FC are included.

**Supporting Table 2: Co-IP-MS analysis of PHF21A-associated proteins in *Phf21a*^+/+^ and *Phf21a*^Δn/Δn^ cortices.**

The complete list of identified proteins in Co-IP-MS comparing *Phf21a*^+/+^ and *Phf21a*^Δn/Δn^ cortices. Raw MS (summed S/N values scaled to 100 per protein) and values and statistics, including -log(Padj), and log2FC are included.

**Supporting Table 3: Functional enrichment analysis of PHF21A-interacting proteins with Metascape.**

Complete list of molecular networks PHF21A-interacting proteins participate. The analysis was done with Metascape(41).

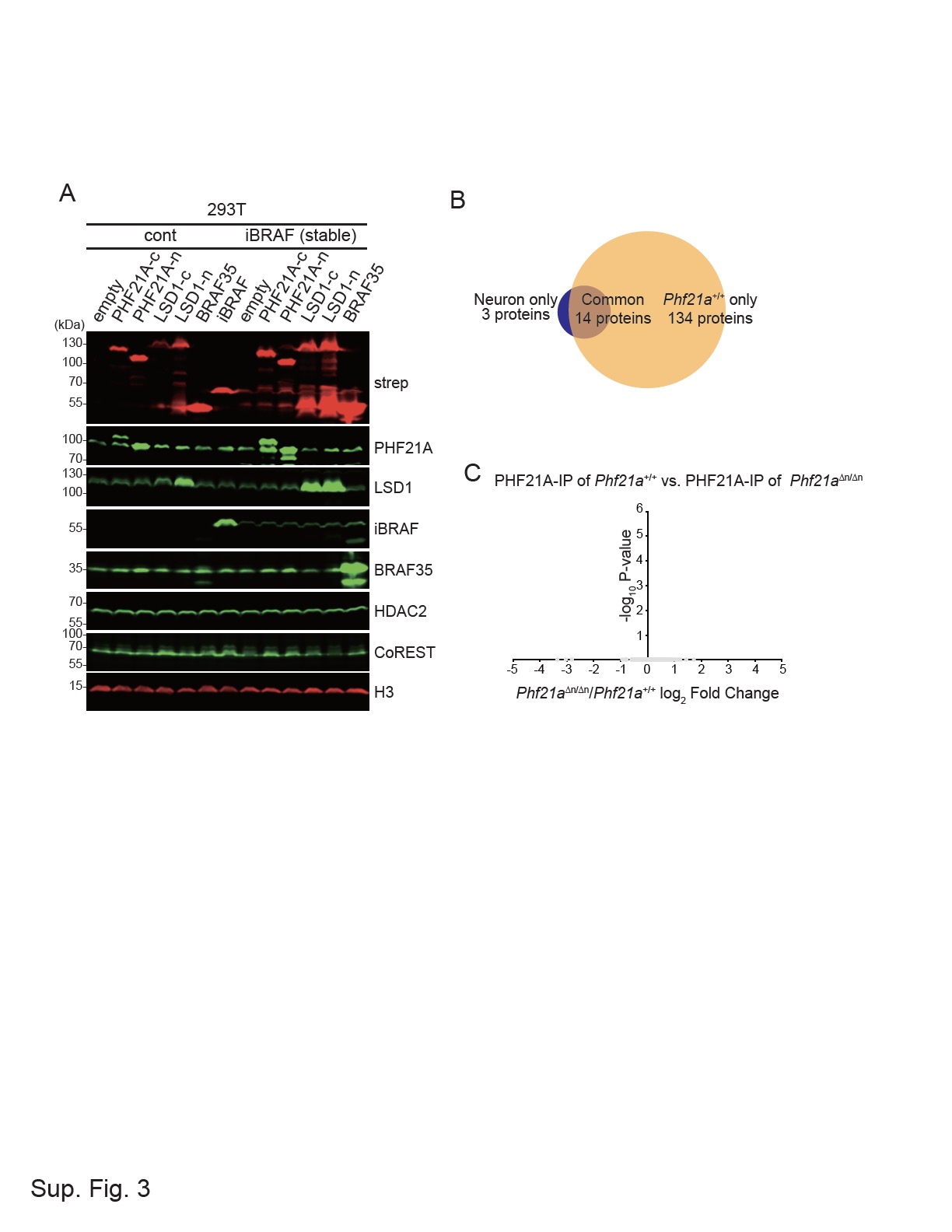

Supplement: Supporting information [file mmc2.docx]
